# A Privacy‐Preserving Markov Chain‐Based Framework for Robust Motor Imagery EEG Classification in Brain–Computer Interfaces

**DOI:** 10.1049/htl2.70093

**Published:** 2026-07-22

**Authors:** Taslima Khanam, Siuly Siuly, Hua Wang

**Affiliations:** ^1^ Institute for Sustainable Industries and Liveable Cities Victoria University Melbourne VIC Australia

**Keywords:** brain–computer interface, electroencephalogram, Markov chain

## Abstract

Accurate classification of motor imagery (MI)‐based electroencephalogram (EEG) signals is often challenged by signal non‐stationarity, subject‐specific variability, and privacy concerns associated with sharing raw neural data. To address these challenges, this study proposes a hybrid Markov chain–spatial statistical (MCSS) machine learning framework for privacy‐preserving MI–EEG classification. Spatially filtered EEG signals were discretised into six symbolic amplitude states representing progressively increasing signal intensities. These symbolic sequences were modelled as stochastic processes, from which transition probability matrices (TPMs) were constructed as the primary feature representation. This TPM‐based abstraction provides a non‐invertible and privacy‐friendly feature space, substantially reducing the ability to reconstruct the original neural waveform. To enhance discriminative performance, the Markov features were combined with seven statistical descriptors. The framework was evaluated using three machine learning algorithms on the brain–computer interface (BCI) Competition III Datasets IVa and IVb. The proposed MCSS + support vector machine framework achieved consistently high classification accuracy (>98%) across all subjects, demonstrating strong robustness and cross‐subject stability. Privacy robustness was further validated through empirical threat‐model evaluation, where membership inference attacks remained close to chance level, and feature inversion attacks showed low reconstruction similarity. Overall, the framework provides an accurate, computationally efficient, and privacy‐preserving solution for scalable BCI development.

## Graphical abstract

We introduce a novel Markov chain (MC)‐based framework for accurate, privacy‐preserving motor imagery task identification in brain–computer interface systems. We model electroencephalogram activity as transitions among six cognitive states. Transition probability matrices are used for feature extraction, providing built‐in privacy protection. Our MC‐support vector machine model achieved >99% accuracy on standard datasets, offering an efficient solution for secure neurorehabilitation applications.

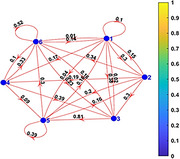

## 1 Introduction

**FIGURE 1:**
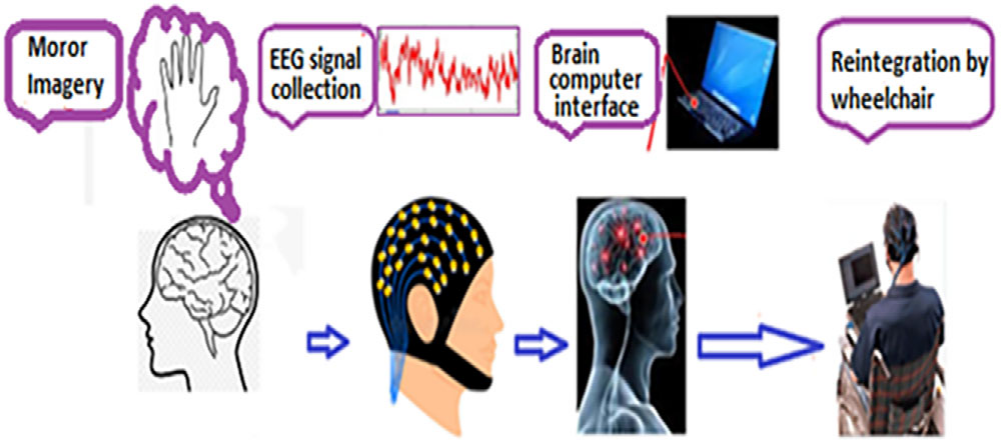
Workflow of MI‐based BCI technique using EEG signal data.

A brain–computer interface (BCI) uses electroencephalogram (EEG) signals as direct input to capture and translate neural activity into control commands for external assistive devices such as robotic arms or wheelchairs. In a typical EEG‐based BCI system, an electrode cap records the user's neural activity, which is subsequently amplified and processed computationally [1, 2, 3, 4, 5, 6, 7]. The most robust signal foundation for a BCI is derived from motor imagery (MI) signals, which involve imagining a movement without physical execution. The generalised data acquisition and processing workflow for MI‐based EEG signals within the BCI framework is shown in Figure 1. Figure 1 shows how an electrode cap measures the brain signals of a person. The electrodes in the cap are linked to a device that amplifies brain waves and records them on EEG signal‐measuring computer equipment. Following EEG signal measurement, BCI decodes the signals, converts them into commands for an output device, like a wheelchair or robotic arm, and carries out the user's intended action [8].

EEG signals are complex time series data containing multiple rhythmic components, including delta, theta, alpha, beta, and gamma waves, each associated with different functional states of the brain [8]. In this study, we focus specifically on processing the delta, theta, alpha, and beta frequency bands within the 0–30 Hz range [9]. The analysis of MI‐based EEG signals typically comprises three main digital signal processing steps: noise removal, feature extraction, and classification to assess MI task performance [10]. Researchers have developed various feature extraction and classification techniques to improve MI‐based EEG recognition accuracy. For signal classification, methods such as spectrogram‐based convolutional neural networks and ensemble support vector machine (SVM) voting schemes have been applied [11, 12, 13]. Common feature extraction techniques often involve Fourier transform, fast Fourier transform, and related frequency‐domain techniques [14]. However, these traditional approaches typically provide static spectral representations and fail to adequately capture the critical temporal evolution of neural activity dynamics during the MI task execution phase.

Several studies have attempted to improve performance through spatial and spatio‐spectral feature learning. Miao et al. (2021) [15] introduced common time–frequency–spatial patterns, achieving a minimum classification accuracy of 52.14% using multiple SVM classifiers. Tiwari and Chaturvedi (2021) [12] reported 98.2% accuracy using common spatial patterns (CSPs) with *K*‐nearest neighbours (KNNs), though CSP‐based spatial filters tend to emphasise class separation but overlook crucial temporal state transitions. Cherloo et al. [16] (2021) applied regularised spatio‐spectral patterns with moderate improvements (68.87% accuracy). Other methods, such as wavelet‐based decomposition and recurrent neural networks, have shown inconsistent performance and often require extensive training or iterative optimisation. Jin et al. (2019) [17] utilised correlation‐based channel selection, reaching 70% accuracy, while Binias et al. (2016) [18] reported 70.24% accuracy using Box–Cox feature normalisation. Djamal et al. (2020) [19] achieved 79.6% accuracy with wavelet–RNN hybrid modelling, though it required numerous iterations. Siuly and Li (2015) [20] applied least‐squares SVM, yet accuracy remained below 80% in the presence of strong subject‐specific signal variability.

Despite these algorithmic advancements, two key signal processing limitations persist: most feature extraction methods do not explicitly model temporal dependencies and dynamic symbolic state transitions fundamental to MI tasks, and many approaches remain sensitive to subject‐specific variability, resulting in inconsistent performance across individual datasets. MI involves evolving neural dynamics across different phases of task engagement. Accurately capturing these evolving quantised transition states is essential for robust MI decoding. To address these technical challenges, we propose a privacy‐preserving hybrid MCSS (Markov chain–spatial statistical) framework that models EEG signals as transitions across six discrete, quantised transition‐relevant states: S1 (Very Low Amplitude State), S2 (Low Amplitude State), S3 (Lower‐Mid Amplitude State), S4 (Upper‐Mid Amplitude State), S5 (High Amplitude State), and S6 (Very High Amplitude State), representing progressively increasing signal intensity levels from the projected EEG responses. Instead of retaining raw EEG waveforms, which contain high‐resolution amplitude patterns and subject‐specific signatures, the framework converts each EEG trial into a transition probability matrix (TPM) that encodes only the probabilistic relationships between symbolic quantised transition states. This abstraction removes fine‐grained temporal waveforms, offering an inherent non‐invertible representation that acts as a privacy‐preserving feature extraction layer. The resulting hybrid MCSS feature representation retains task‐distinguishing dynamics while intrinsically suppressing subject‐specific neural signatures, thereby enhancing data security and robustness.

By transforming the continuous EEG signals into generalised state‐transition probabilities, the framework enhances adaptability to individual EEG patterns and enables efficient real‐time classification. The extracted features were evaluated using three widely adopted machine learning models: SVM, KNN, and decision tree (DT) to benchmark generalisability across subjects. To the best of our knowledge, this is the first work to apply a six‐state Markov chain (MC) representation to MI‐based EEG for robust decoding and non‐invertible feature abstraction. The three major contributions of this research are as follows:

1. Development of a privacy‐preserving and robust AI‐based framework for MI‐based BCI systems, enabling reliable interpretation of MI signals while substantially reducing exposure of raw neural activity patterns.
2. Introduction of a novel privacy‐preserving hybrid MCSS feature extraction framework, in which EEG signals are transformed into discrete state‐transition representations that capture temporal dynamics and complementary statistical characteristics while minimising subject‐identifiable waveform information.
3. Comprehensive comparative and privacy robustness evaluation of the proposed MCSS feature representation, including multiple baseline classifiers (SVM, KNN, and DT); supplementary deep learning benchmarking (EEGNet); comparative wavelet‐based preprocessing analysis; and two empirical privacy threat models (likelihood‐based membership inference attack (MIA) and feature inversion attack) to assess generalisability, robustness, and privacy preservation across different subjects.

The remainder of this paper is organised as follows. Section 2 describes the dataset and the proposed privacy‐preserving hybrid MCSS framework; Section 3 presents the experimental results; Section 4 provides discussion and comparative analysis; and Section 5 concludes the study.

## 2 Methodology

The proposed framework begins by applying a fifth‐order Butterworth band‐pass filter (1–30 Hz) to the MI‐based EEG signals to suppress noise and preserve MI–relevant neural oscillations. Raw EEG trials were segmented using cue onset markers with a fixed 5‐s analysis window following cue onset (0.001–5.001 s). In the supplementary analysis, a wavelet‐based denoising pipeline was additionally implemented for comparison. Following preprocessing, a privacy‐preserving hybrid MCSS feature extraction framework was applied. Within each training fold, covariance matrices were computed to learn subject‐specific spatial filters, and the projected EEG amplitudes were discretised into six symbolic amplitude states (S1–S6) using predefined quantisation thresholds. Based on these symbolic sequences, a 6 × 6 TPM was constructed for each trial and subsequently flattened into a 36‐dimensional Markov feature vector.

**FIGURE 2:**
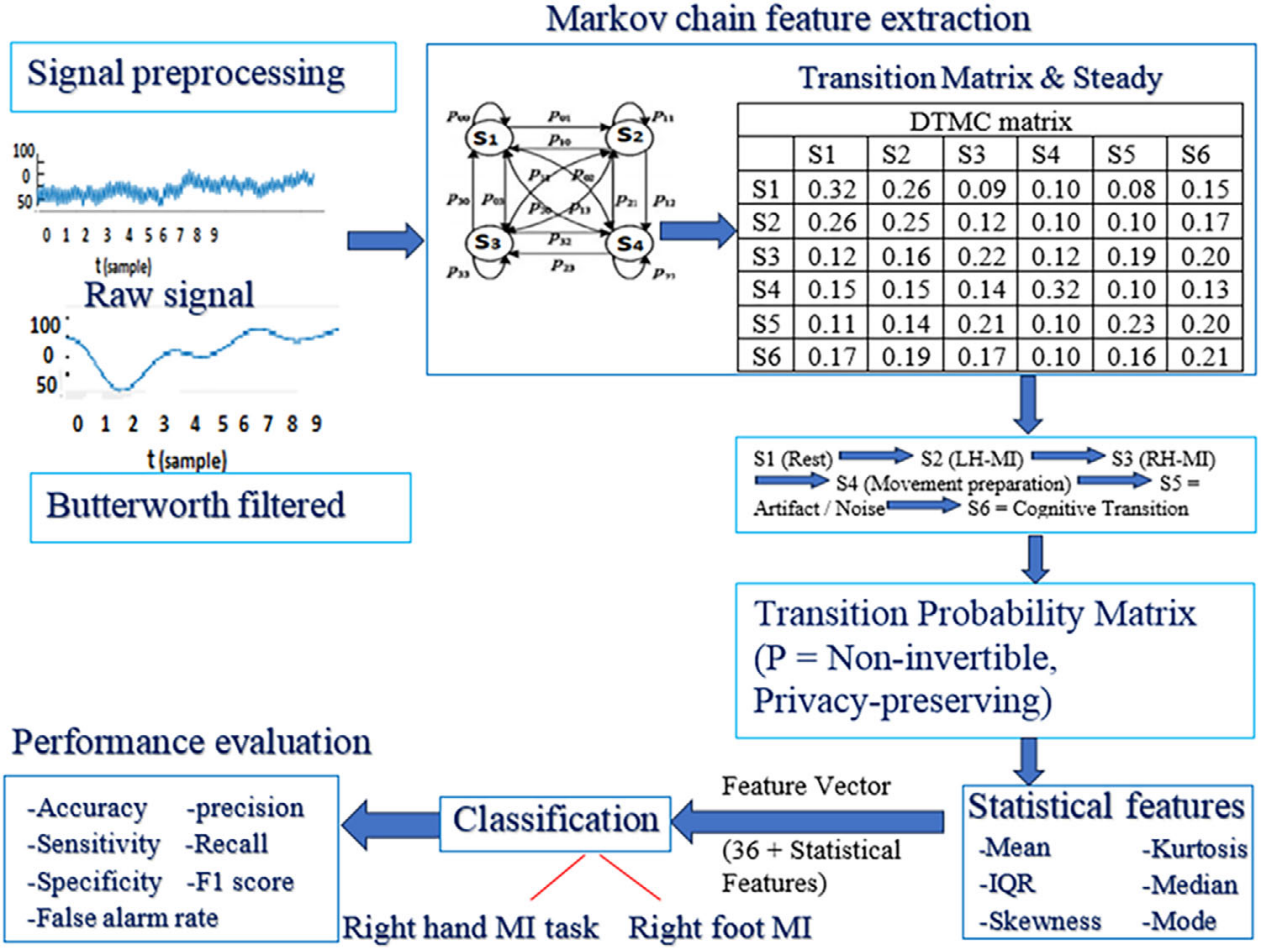
Flowchart of the proposed technique for identifying MI tasks using EEG signal classification in BCI development.

To enrich the representation while preserving privacy, a complementary 120‐dimensional statistical feature vector was extracted from the spatially projected EEG components, including mean, variance, skewness, kurtosis, median, interquartile range, and related distributional descriptors. For the combined framework, Markov, statistical, and CSP‐derived spatial log‐variance features were concatenated into a unified feature space prior to classification. To ensure full reproducibility and leakage prevention, all feature construction, parameter tuning, normalisation, and classifier fitting were performed strictly within the training portion of each cross‐validation fold, and the selected optimal parameters were subsequently evaluated on the corresponding held‐out test fold. This non‐invertible TPM‐based abstraction inherently reduces subject re‐identification risk while maintaining high classification accuracy. The methodological framework is shown in Figure 2.

### 2.1 Dataset

The proposed method was assessed using EEG data from the publicly available BCI Competition III Datasets IVa and IVb [21]. The dataset (BCI competition III dataset IVa) consists of multichannel EEG recordings obtained from five healthy participants seated comfortably in a relaxed chair with their arms supported on armrests. A total of 118 EEG channels were recorded for each subject. According to the original dataset documentation, the EEG signals were initially band‐pass filtered within the frequency range of 0.05–200 Hz, acquired at a sampling frequency of 1000 Hz, and subsequently down‐sampled to 100 Hz for analysis. An inter‐trial interval of approximately 2 s was incorporated to allow participants a brief rest between consecutive trials. For each subject, the dataset contains 280 trials, with all 118 channels recorded throughout each trial. Two MI tasks collected in this dataset were right‐hand MI (RH‐MI) and right‐foot MI (RF‐MI). The dataset (BCI Competition III Dataset IVb) comprises multichannel EEG recordings collected from a healthy participant performing MI tasks while seated in a relaxed position. The dataset includes 118 EEG channels, with the signals initially band‐pass filtered between 0.05 and 200 Hz. Data acquisition was performed at a sampling frequency of 1000 Hz, followed by down‐sampling to 100 Hz for subsequent analysis. A brief inter‐trial interval of approximately 2 s was maintained to provide short resting periods between trials. In total, the dataset contains 210 trials, with all 118 EEG channels recorded for each trial. Two MI tasks collected in this dataset were left‐hand MI (LH‐MI) and RF‐MI. Due to the highly subject‐specific dependence of MI‐based EEG signals on physical activities, the proposed methods’ experiments in both datasets were carried out for each subject individually. For reproducibility, the full stepwise implementation of the proposed framework is summarised in Algorithm 1.

ALGORITHM 1 **Proposed Privacy‐Preserving MC‐SVM Pipeline.**

**Table:** 

Input: Pre‐segmented MI‐EEG trials	
Output: Classified MI labels	
1.	Bandpass filter EEG (1–30 Hz)
2.	Compute normalised trial covariance matrices
3.	Estimate class‐wise covariance matrices
4.	Perform whitening and spatial projection
5.	Project EEG trials onto spatial filters
6.	Quantise projected amplitudes into 6 states
7.	Construct 6 × 6 TPM
8.	Flatten TPM → 36‐dimensional Markov features
9.	Extract 120 statistical descriptors
10.	Concatenate all features
11.	Normalise using training‐fold *z*‐score statistics
12.	Perform SVM classification

### 2.2 Signal Pre‐Processing

Pre‐processing is a critical step to reduce noise and improve the quality of EEG signals before analysis. EEG signals are inherently prone to contamination due to artefacts such as eye blinks, muscle movement, and electrical interference. In this study, a fifth‐order Butterworth bandpass filter was applied with cutoff frequencies of 1 Hz (low) and 30 Hz (high). The Butterworth filter offers a maximally flat frequency response in the passband, minimising distortion.

The filter equation in the time domain is given by

(1) $$y\left(n\right)=\sum_{i=0}^{N}\left(a_{i}x\left(n-i\right)\right)+\sum_{j=1}^{N}\left(b_{i}y\left(n-j\right)\right)\;$$

The corresponding *z*‐transform representation is

(2) $$H\left(z\right)=\frac{\sum_{i=0}^{N}a_{i}z^{-i}}{1+\sum_{j=1}^{N}b_{j}z^{-i}}\;$$

where *N* is the filter order, $a_{i}$ and $b_{j}$ are the filter coefficients, x(n) is the input signal, and y(n) is the output signal.

To address the robustness of the proposed framework against alternative denoising strategies, a supplementary preprocessing comparison was conducted using a discrete wavelet transform–based denoising pipeline. Specifically, a Daubechies wavelet (db4) with level‐2 decomposition was applied, and MI–relevant detail coefficients were reconstructed prior to hybrid MC‐statistical feature extraction. This supplementary analysis was included to compare the proposed Butterworth‐based preprocessing with an automated time–frequency denoising approach.

### 2.3 Privacy‐Preserving Hybrid MCSS Feature Framework

Following noise removal, we applied the proposed hybrid MCSS feature framework. The methodological novelty lies in the TPM‐based Markov representation and its integration with complementary statistical descriptors extracted from the spatially filtered EEG projections. In contrast, covariance computation, whitening, spatial filtering, and conventional classifiers are employed as baseline processing components. This hybrid design enables simultaneous modelling of temporal transition dynamics and distributional signal characteristics while preserving privacy through non‐invertible abstraction.

#### 2.3.1 Covariance Matrix Construction

For each trial *t*, the normalised covariance matrix is computed as

(3) $$\;\sum_{t}=\frac{E_{t}E_{t}^{T}}{\mathrm{trace}\;\left(E_{t}E_{t}^{T}\right)}\;$$

where $E_{t}\in R^{C\times T}$ is the EEG signal matrix (*C* = 118 channels, *T* = time samples).

This normalisation ensures scale invariance across trials.

#### 2.3.2 Class‐Wise Covariance Aggregation

Class‐wise average covariance matrices are computed for left‐hand and right‐foot imagery

(4) $$\;\sum_{\mathrm{total}}=\sum_{\mathrm{class}1}+\sum_{\mathrm{class}2}\;$$

#### 2.3.3 Whitening and Projection Matrix Construction

We perform eigenvalue decomposition of $\sum_{\mathrm{total}}\mathrm{yields}$

$$\;\sum_{\mathrm{total}}=\;UDU^{T}$$

The whitening matrix is computed as

(5) $$P=D^{\frac{-1}{2}}U^{T}$$

This step decorrelates spatial channels and enables separation of class‐discriminative projections.

The spatial filters *W* are then obtained by

(6) $$W={\left[U_{1}\;U_{2}\right]}^{T}$$

where $U_{1}$ and $U_{2}$ are eigenvectors corresponding to the dominant components of each MI class.

The resulting projected signals are subsequently used for Markov‐based state transition modelling. Each trial's spatially filtered data is discretised into symbolic states using amplitude‐based quantisation.

#### 2.3.4 Discretisation into Six Quantised Transition States (S1–S6)

Based on spatio‐temporal EEG projections, the continuous signal amplitudes were discretised into six ordered quantised transition states (S1–S6) using an amplitude‐based quantisation scheme. These symbolic states were derived from the spatially filtered EEG trial responses and associated statistical projections, where the continuous signal range was partitioned into six ordered intervals using predefined quantisation boundaries. Each projected EEG sample was then assigned to one of the six symbolic states according to its corresponding amplitude level. The six symbolic states were defined as S1 (very low amplitude state), S2 (low amplitude state), S3 (lower‐mid amplitude state), S4 (upper‐mid amplitude state), S5 (high amplitude state), and S6 (very high amplitude state), representing progressively increasing signal intensity levels from the projected EEG responses. These states should be interpreted as quantised signal levels rather than direct physiological or cognitive states, and their primary purpose is to preserve the temporal transition dynamics of the EEG signal for hybrid MCSS feature extraction. Each filtered EEG segment is labelled into one of these six states by applying value binning over projection magnitudes:

$X_{t}:\left(x_{1},\;x_{2},\text{…}\;x_{s}\right);\;X_{t}:\left(x_{i}\right)$, where *i* = 1, 2,….*s*.

(7) $$x_{i}\in \left[q_{\mathrm{Low}}+\left(k-1\right)*\mathrm{step},\;\;q_{\mathrm{Low}}+k*\mathrm{step}\right]$$

where $q_{\mathrm{Low}}=\left(\min\left(X_{t}\;\right)-\Delta {\bar{X}}_{t}\right)$ is the lower limit of the time series, $q_{\mathrm{up}}=\left(\max\left(X_{t}\;\right)+\Delta {\bar{X}}_{t}\right)$ is the upper limit of the time series, and step =$\left(q_{\mathrm{up}}\;-q_{\mathrm{Low}}\right)/k_{\max}$, *k* ∊ {1, 2, 3, 4, 5, 6} denotes the assigned state index.

**FIGURE 3:**
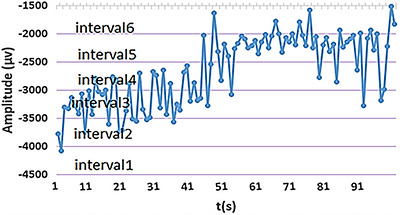
An example of the Markov chain generation process of subject *aa*.

An example of six states of the MC‐generating process in the EEG dataset is shown in Figure 3. A 100‐length time series was transformed into a six‐state MC using the transform rule, and the resulting pattern is shown in this figure.

#### 2.3.5 Transition Probability Matrix Construction

From each sequence of states per trial, we count transitions between states to build a transition frequency matrix

$D\in R^{6\;\times \;6}$: $D_{ij}=$ count of transitions from $S_{i}$ and $S_{j}$

This is normalised to form the TPM

(8) $$P_{ij}\;=\;\frac{D_{ij}\;}{\sum_{j=1}^{6}D_{ij}\;}$$

Flattening the TPM gives the Markov feature vector

(9)

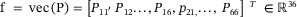




Since multiple distinct EEG signals can produce the same TPM, the mapping from EEG to *P* is non‐invertible and, by design, substantially reduces the ability to reconstruct the original neural activity patterns. This property provides intrinsic privacy preservation in the proposed framework. Compared with conventional waveform‐level representations, the proposed hybrid abstraction offers a privacy‐friendly feature representation with reduced invertibility, as supported by the empirical MIA and inversion analyses.

#### 2.3.6 Privacy‐Preserving Property (Key Advantage)

By converting EEG signals into discrete state sequences and representing each trial only through its state‐transition probabilities, the proposed method removes identifiable temporal and spatial waveform characteristics that can reveal personal neural signatures. Since TPMs capture only transition dynamics and not raw signal shapes, the original EEG cannot be reconstructed:

EEG →/*P* ⇒ Non‐invertible representation

In this formulation, *P* denotes the TPM derived from symbolic quantised transition states; because P contains only aggregated transition dynamics rather than raw EEG waveforms, the transformation is non‐invertible, substantially reducing reconstruction risk and thereby enhancing privacy preservation.

### 2.4 Statistical Feature Extraction

Following spatial projection, statistical descriptors were computed for each retained spatial component on a per‐trial basis. Specifically, for every individual trial, the projected EEG signal from each retained spatial filter was summarised using six distributional descriptors: mean, skewness, kurtosis, median, mode, and interquartile range (IQR = Q3 − Q1) [22]. A total of 20 spatial filters were retained (10 from each class); therefore, the statistical feature extraction stage produced 6 × 20 = 120 features per trial. These component‐wise descriptors were subsequently concatenated across all retained filters to form a 120‐dimensional trial‐level statistical feature vector. When combined with the corresponding class label, each trial was represented as a 121‐dimensional labelled feature vector for supervised learning. Within the proposed hybrid MCSS framework, these statistical descriptors serve as a complementary feature branch that augments the TPM‐based Markov transition representation, resulting in a three‐stage architecture comprising (i) baseline spatial filtering, (ii) Markov transition‐based feature extraction, and (iii) statistical feature integration.

### 2.5 Classification

The classifiers were used as comparative baseline learners to evaluate the discriminative effectiveness of the proposed hybrid feature representation. To evaluate the discriminative performance of the proposed framework, three widely used supervised machine learning classifiers were employed: SVM, KNN, and DT. All experiments were implemented in MATLAB (R2024b; MathWorks, USA) using the Statistics and Machine Learning Toolbox and Wavelet Toolbox, ensuring reproducibility of preprocessing, feature extraction, and classification procedures under a leakage‐safe stratified 10‐fold cross‐validation protocol, where approximately 90% of the trials were used for training and 10% for testing in each fold.

#### 2.5.1 Support Vector Machine

The SVM classifier was configured using a radial basis function kernel, with kernel scale set to automatic estimation and box constraint fixed at 1. Prior to model fitting, all features were standardised using *z*‐score normalisation based exclusively on the training fold statistics, and the same normalisation parameters were subsequently applied to the corresponding test fold. Our research focused on the two‐class feature classification, and computational interpretation can be termed as follows:

(10) $$c\left(f\right)=\mathrm{sign}\left(\sum_{j=1}^{n}\alpha_{j}y_{j}K\left({x,x}_{j}\right)+b\right.$$

where x is the test feature vector, $x_{j}$ is the j‐th training/support vector $y_{j}\in -1,+1$ is the corresponding class label, and $\alpha_{j}$ is the Lagrange multipliers, K(·,·) is the kernel function, bis the bias term, and nis the number of support vectors or training samples used in the decision function [23]. In this study, SVM was applied for binary MI classification.

#### 2.5.2 K‐Nearest Neighbor

Using a similarity metric to classify new data or cases, KNN is a straightforward algorithm that stores all of the previously classified cases. The main application of it is to categorise data points based on the classification of their neighbors. Based on feature similarity, the value of *K* in the KNN algorithm represents the number of nearest neighbors used in the majority voting process. To increase accuracy, it is crucial to choose the right value for *K*, which calls for parameter modification. This classifier has the benefits of being straightforward; being flexible in terms of features; easily handling situations involving multiple classes; and requiring sizable datasets. Both the requirement for a meaningful distance between neighbours and the high computation cost are drawbacks of the KNN classifier [24]. In this study, the KNN classifier was configured with *K* = 50 nearest neighbours (This value was selected based on fold‐wise validation within the training data) using the default Euclidean distance metric, serving as a simple non‐parametric baseline for comparison. Similar train‐fold‐only *z*‐score normalisation was applied before classification.

#### 2.5.3 Decision Tree

An algorithm known as a DT is used for classification and regression tasks in non‐parametric machine learning. A root node, branches, a minimum number of observations per node, internal nodes, and leaf nodes make up its hierarchical tree structure. A DT has a root node at the beginning that has no incoming branches. The internal nodes, also referred to as decision nodes, are fed by the root node's outgoing branches. Both types of node conduct assessments based on the features, which are represented by leaf or terminal nodes. The dataset's leaf nodes represent all outcomes that could possibly occur. In this study, leaf nodes = 1 and the minimum number of observations per node = 10; other parameters were constant. DT classifiers have the advantages of being straightforward to comprehend and interpret, requiring little data preparation, and being able to handle both categorical and numerical data. The drawbacks, however, include a tendency for data resampling, overfitting, and feature reduction [25]. A standard binary DT classifier was used with maximum splits = default and minimum leaf size = 1 provided by the machine learning toolbox. Input features were standardised within each training fold before model fitting.

#### 2.5.4 Deep Learning Comparative Analysis

To provide broader methodological context and address reviewer feedback, supplementary deep learning experiments, a statistical‐only baseline, CSP‐based baseline, and combined feature framework were performed. EEGNet was selected as the deep learning benchmark because it is one of the most widely adopted EEG‐specific convolutional neural network architectures and has demonstrated strong performance across a wide range of BCI and MI EEG classification studies. Furthermore, EEGNet was specifically designed for EEG signal analysis and provides a compact architecture suitable for relatively small EEG datasets. Therefore, EEGNet was considered an appropriate representative deep learning comparator for evaluating the proposed MCSS framework. All deep learning experiments were conducted using EEGNet on the same hybrid MC‐statistical feature space. The statistical‐only framework was based on conventional handcrafted EEG descriptors extracted from each trial, including central tendency and dispersion measures (e.g., mean, variance, standard deviation, and percentile‐based statistics), and served as a non‐transition‐based baseline for comparison. The CSP baseline was implemented using four filter pairs (eight spatial components), and log‐variance features were extracted from the projected EEG trials prior to classification. The combined framework integrates three complementary feature representations: Markov transition features, conventional statistical descriptors, and CSP‐derived spatial log‐variance features, which were concatenated into a unified feature vector prior to classification. These analyses were included as comparative benchmarks rather than replacements for the primary machine learning framework.

#### 2.5.5 Privacy Threat Model and Empirical Privacy Evaluation

To assess the privacy‐preserving properties of the proposed MCSS‐based feature representation, two empirical adversarial threat models were considered: likelihood‐based MIA and feature inversion attack. These experiments were designed to evaluate whether the extracted transition‐based features could leak membership information or allow reconstruction of the original EEG signal.

1. Threat model definition
  An honest‐but‐curious adversary was assumed, where the attacker has access to the released MCSS feature vectors and corresponding model outputs but does not have direct access to the original raw EEG signals. The objective of the attacker is twofold:
  - determine whether a specific sample was used during model training (membership inference),
  - reconstruct the original EEG representation from the released features (feature inversion).
2. Likelihood MIA methodology
  For the MIA, a likelihood‐based MIA framework was implemented. Specifically, the attacker utilised the classifier's output confidence/decision scores for member and non‐member samples to estimate membership likelihood [26]. Attack performance was quantified using attack accuracy (%) and area under the ROC curve (AUC). An attack accuracy close to 50% and AUC close to 0.50 were interpreted as chance‐level inference, indicating minimal membership leakage.
3. Feature inversion methodology
  To evaluate feature invertibility, a feature inversion attack was performed in which an adversarial regression model was trained to reconstruct the original EEG signal from the released hybrid features [27]. The inversion quality was assessed using mean squared error (MSE) and the Pearson correlation coefficient between the reconstructed and original EEG representations. Higher reconstruction error and lower correlation indicate stronger resistance to inversion and improved privacy preservation.

#### 2.5.6 Evaluation Protocol

All experiments were conducted on a per‐subject basis using a leakage‐safe stratified 10‐fold cross‐validation protocol. For each subject, trials were partitioned into 10 stratified folds preserving class balance. In each iteration, nine folds (approximately 90%) were used for training and one fold (approximately 10%) for testing. All preprocessing steps, including covariance estimation, spatial filter learning, state discretisation thresholds, feature scaling, and classifier fitting, were performed exclusively on the training folds and subsequently applied to the held‐out test fold. No information from the test fold was used during feature extraction, normalisation, or model fitting. Classifier hyperparameters were selected through fold‐wise tuning within the training data and kept fixed during evaluation on the corresponding test folds. For reproducibility, the full stepwise implementation of the privacy threat model and attack evaluation is summarised in Algorithm 2.

ALGORITHM 2 **Privacy Threat Model and Attack Evaluation.**

**Table:** 

Input: Markov‐derived feature vectors and classifier outputs	
Output: Privacy leakage metrics	
1.	Define honest‐but‐curious adversary model
2.	Assume attacker has access to:
a. released Markov feature vectors	
b. classifier confidence scores/decision outputs	
3.	Perform Membership Inference Attack:
a. separate member and non‐member samples	
b. compute membership likelihood scores	
c. estimate attack accuracy	
d. compute ROC‐AUC	
4.	Perform Feature Inversion Attack:
a. train adversarial regression model	
b. reconstructs approx. EEG representation	
c. compute reconstruction MSE	
d. compute Pearson correlation	
5.	Report privacy metrics:
attack accuracy, AUC, MSE, correlation	
6.	Interpret privacy risk:
chance‐level MIA and low correlation indicate privacy preservation

## 3 Results

In this study, we evaluated the proposed privacy‐preserving hybrid MCSS framework for MI EEG classification using SVM, KNN, and DT classifiers under a leakage‐safe stratified 10‐fold cross‐validation protocol. All feature extraction, normalisation, and classification steps were performed strictly within each training fold and subsequently applied to the corresponding test fold to minimise overfitting and information leakage. The results presented below summarise the classification performance, comparative benchmarking across preprocessing and classifier settings, and the privacy robustness of the proposed framework.

**FIGURE 4:**
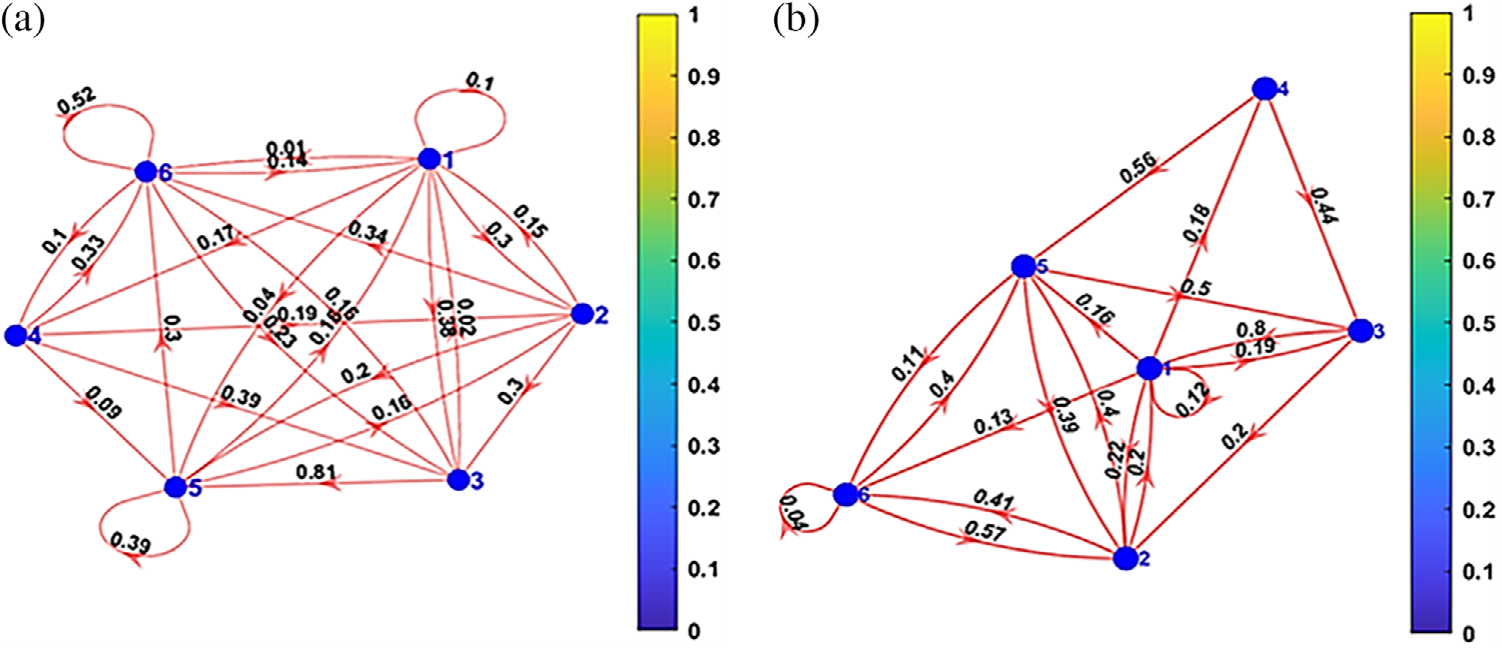
Functional systems for every state as well as predicted probabilities of transition between states for subject aa of dataset IVa (a) and dataset IVb (b).

Figure 4 illustrates the six‐state quantised transition dynamics for subject *aa* across Dataset IVa (Figure 4a) and Dataset IVb (Figure 4b). Figure 4a demonstrates that subject aa exhibits strong state persistence, particularly in the higher amplitude symbolic states (S5–S6), together with highly structured inter‐state transitions. These stable transition dynamics are consistent with the strong discriminative performance of the proposed MCSS + SVM framework. Figure 4b exhibits clearer and more directional transitions (e.g., S2 → S3 and S3 → S4). A dominant transition is observed from S6 to S3 ($P_{63}\;=0.80$), indicating frequent movement from the very high amplitude state to the lower‐mid state. Strong transitions are also seen from S5 to S4 ($P_{54}=0.56$) and S5 to S2 ($P_{52}=0.39),$ suggesting dynamic shifts between higher and moderate signal levels. In addition, S3 to S4 shows a notable transition probability ($P_{34}\;=0.44$), while the self‐transition of S1 remains low ($P_{11}\;=0.06$), indicating limited persistence in the lowest state. Overall, the strong inter‐state transitions, particularly S6→S3, demonstrate stable temporal dynamics that support the high classification accuracy of the proposed MCSS+SVM framework.

**FIGURE 5:**
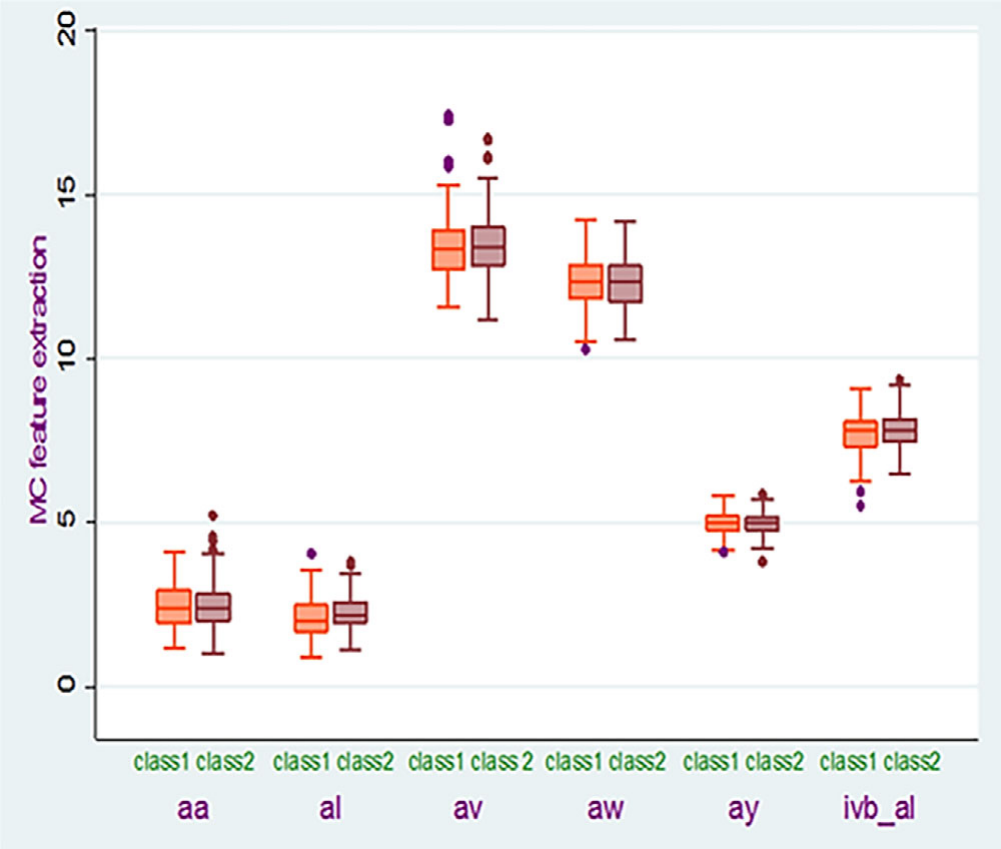
Boxplot of all subjects considering two classes by applying the MC feature extraction method.

Figure 5 presents boxplots of MC‐derived feature distributions for two MI classes across six subjects (aa, al, av, aw, ay, and ivb). Subjects aa, al, and ay display clearly separated feature distributions between classes, indicating strong discriminability. Subject av shows higher variance and outliers, likely reflecting increased neural variability or noise. Subjects aw and ivb also exhibit distinguishable class patterns. Overall, the consistent separation across subjects confirms that the MCSS feature representation preserves meaningful class‐related information, despite abstracting the original EEG into non‐invertible transition statistics. Importantly, because the TPM‐based representation aggregates only transition structure and does not retain continuous waveform information, it provides an inherent privacy‐preserving property while still enabling high classification performance. This confirms that privacy‐preserving feature abstraction and accurate MI decoding can be achieved simultaneously.

### 3.1 Parameter Selection

A fold‐wise parameter tuning was conducted to evaluate the effect of sample length (*S*), boundary value (Δ), and number of symbolic states (*N*) on classifier performance. Table 1 represents parameter tuning accuracy (%) for KNN, SVM, and DT classifiers under varying MCSS feature extraction settings. It highlights the impact of varying *S* = sample lengths (500, 300, and 200), revealing that SVM consistently outperforms KNN and DT, particularly with the longest sample length of 500, where it achieves near‐perfect accuracy for most subjects. As the sample length decreases, SVM's performance declines noticeably, while DT maintains relatively stable accuracy, and KNN remains the least accurate overall. It also explores the influence of ∆ = boundary values (0.001, 0.005, and 0.0001), showing that SVM yields the highest and most consistent results at a boundary of 0.001, indicating its sensitivity to tuning parameters. KNN and DT display mixed trends across boundary values, without a consistent pattern. From Table 1, when *S* = 500, ∆ = 0.001, and *N* = 6, most subjects performed at their best levels. When *N* = 5, subjects aa and aw displayed their best performance.

**TABLE 1:** Parameter tuning accuracy (%) for KNN, SVM, and DT classifiers under varying MCSS feature extraction settings.

Sub	BV	*S* = 500, *N* = 6			*S*	∆ = 0.001 and *N* = 6			# State	∆ = 0.001 and *S* = 500		
		KNN	SVM	DT		KNN	SVM	DT		KNN	SVM	DT
aa	0.001	75.35	99.6	97.6	500	75.35	99.6	97.6	5	75.35	99.6	97.6
	0.005	66.7	86.7	90.5	300	62.5	89.9	87.5	4	67.3	76.8	87.5
	0.0001	69.6	90.5	88.7	200	63.1	75.6	87.5	6	64.9	83.3	88.1
al	0.001	62.9	100	87.10	500	62.9	100	87.1	6	62.9	100	87.1
	0.005	67.3	95	88.7	300	73.8	76.8	88.7	4	67.9	76.2	85.1
	0.0001	70.2	93.1	86.3	200	70.2	74.4	71.4	5	66.1	82.7	81.5
av	0.001	58.1	99.2	90.5	500	58.1	99.2	90.5	6	58.1	99.2	90.5
	0.005	66.7	94	85.7	300	57.1	95.2	83.3	4	63.1	86.9	84.5
	0.0001	63.1	93.8	71.4	200	59.5	70.2	71.4	5	66.7	98.8	82.1
aw	0.001	58.9	99.3	89.3	500	58.9	99.3	89.3	6	55.4	99.3	87.3
	0.005	58.9	89.3	91.1	300	67.9	85.7	85.7	5	58.9	99.3	89.3
	0.0001	57.1	96.4	82.1	200	57.1	96.4	82.1	4	57.1	96.4	71.4
ay	0.001	64.3	98.57	92.9	500	64.3	98.57	92.9	6	64.3	98.57	92.9
	0.005	64.3	82.1	89.3	300	64.3	92.9	92.9	5	85.7	53.6	89.3
	0.0001	67.9	78.6	78.6	200	60.7	89.3	89.3	4	64.3	89.3	92.9
IVb	0.001	68.1	99.5	88.6	500	68.1	99.5	88.6	6	68.1	99.5	88.6
	0.005	61.9	87.6	90	300	64.8	85.7	89.0
	0.0001	66.2	85.7	87.6

From Table 2, under the optimal parameter setting (BV = 0.001, sample length = 500, *N* = 6), SVM consistently outperformed DT and KNN across all subjects, achieving near‐perfect values for all performance metrics and a zero false alarm rate. DT showed stable but comparatively lower performance, while KNN remained the weakest classifier. These findings confirm that the proposed MC–SVM framework provides highly accurate and reliable MI EEG classification. These findings further support the selection of SVM as the primary classifier for the proposed privacy‐preserving MCSS + SVM framework.

**TABLE 2:** Performance metrics of KNN, SVM, and DT classifiers under the optimal MCSS feature extraction setting (BV = 0.001, sample length = 500, *N* = 6).

Subject	Classifier	Sensitivity	Specificity	Precision	Recall	F1 score	False alarm rate
aa	SVM	100	99.29	99.29	100	99.64	0
	DT	91.25	84.02	81.25	92.03	86.09	18.75
	KNN	74.01	58.29	74.21	59.11	0.65	58.75
al	SVM	100	100	100	100	100	0
	DT	85.1	89.09	90.01	85.21	87.01	9.82
	KNN	50.24	89.08	81.22	70.11	69.29	18.75
av	SVM	98.57	100	100	98.57	99.28	0
	DT	87.04	82.2	88.03	87.21	85.02	19.04
	KNN	59.21	70.17	81.3	70.13	69.08	19.04
aw	SVM	99.29	100	100	99.29	99.64	0
	DT	92.21	85.07	87.01	93.13	90	13.33
	KNN	58.06	64.03	87.21	64.01	69.06	13.33
ay	SVM	97.86	99.29	99.28	97.86	98.57	0.71
	DT	93.11	75.1	90.01	94.11	88.01	0
	KNN	64.21	88.31	100	64.21	78.02	0
IVb	SVM	99.52	100	99.06	100	99.53	0.95
	DT	92.01	85.05	93.11	93.02	89.07	16.19
	KNN	63.03	80.13	88.21	80.01	73.01	12.38

**FIGURE 6:**
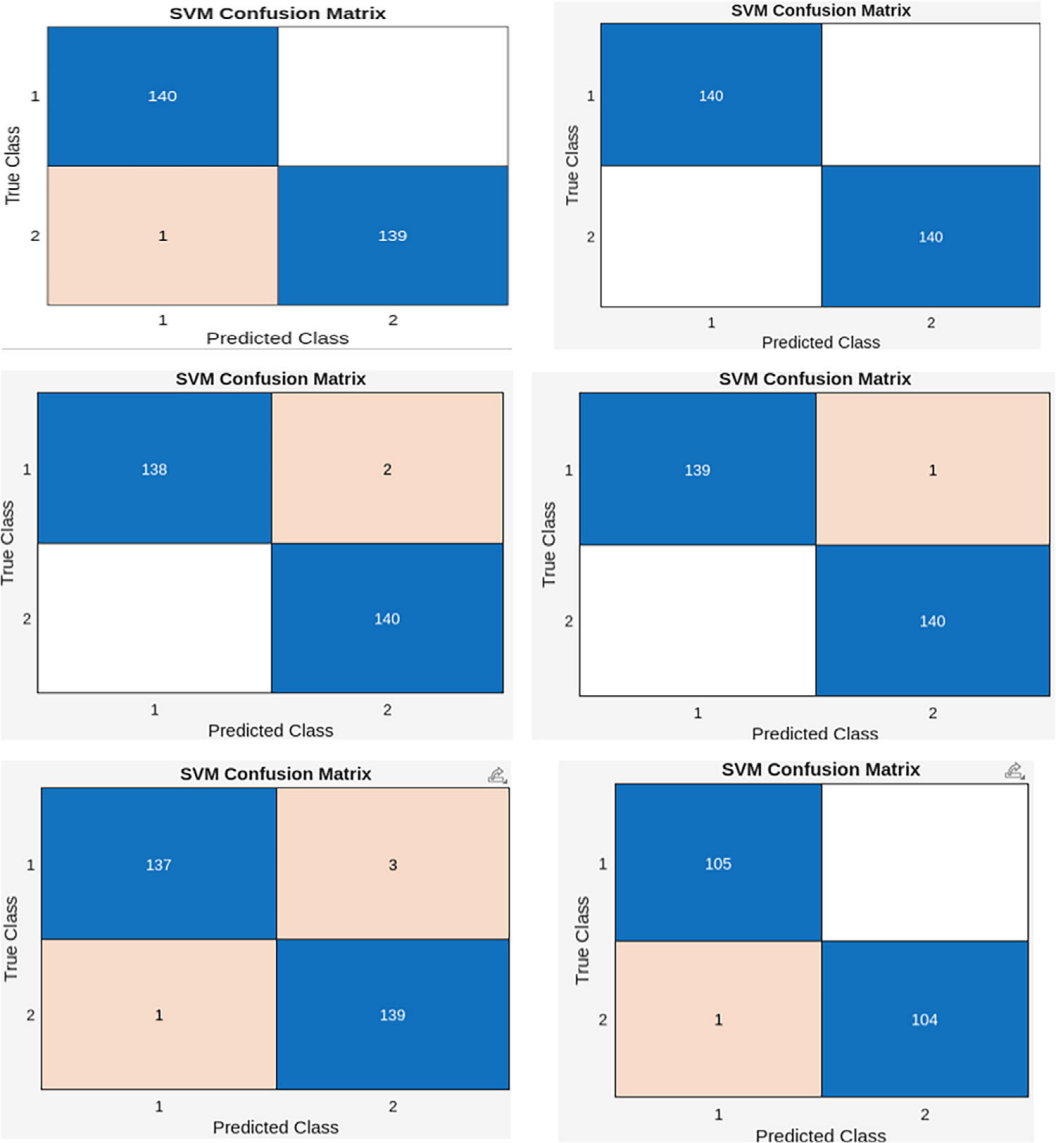
Confusion matrices of the proposed MCSS + SVM framework for all six subjects aa, al, av, aw, ay, and IVb, respectively, under leakage‐safe stratified 10‐fold cross‐validation. For subjects aa, al, av, aw, and ay, Class 1 corresponds to RH‐MI, and Class 2 corresponds to RF‐MI, whereas for subject IVb, Class 1 stands for LH‐MI and Class 2 stands for RF‐MI.

Figure 6 shows the confusion matrices for subjects aa, al, av, aw, ay, and IVb, respectively. All matrices demonstrate strong diagonal dominance with minimal off‐diagonal misclassification, confirming excellent class separability and supporting the high performance metrics reported in Table 2.

Table 3 presents the fold‐wise ablation and comparative classification accuracy of the proposed framework and benchmark feature extraction pipelines across all subjects under both Butterworth and wavelet‐based preprocessing. The CSP + SVM baseline was included as the primary protocol‐matched conventional benchmark, while EEGNet served as the modern deep learning comparator. Under Butterworth preprocessing, the proposed MCSS + SVM framework achieved the highest classification performance across all subjects, with accuracies ranging from 98.57% to 100.00%, including a perfect accuracy for subject al (100.00 ± 0.00%). Similarly high performance was observed for subjects aa (99.60 ± 1.12%), aw (99.64 ± 1.13%), and IVb (99.52 ± 1.51%). Under wavelet‐based preprocessing, the proposed framework also maintained consistently high performance, with accuracies ranging from 96.79% to 99.05%, indicating strong stability across alternative denoising strategies.

**TABLE 3:** Ablation and comparative performance analysis of feature extraction frameworks across preprocessing strategies.

Methods		Accuracy (%)					
		Signal preprocessing with a Butterworth filter					Wavelet
		Proposed framework (MCSS + SVM)	MCSS + EEGNet	Statistical + SVM	Markov + SVM	CSP + SVM	Proposed framework
Subject	aa	99.6 ± 1.12	98.57 ± 2.50	56.07 ± 6.31	97.50 ± 3.39	63.93 ± 10.71	98.57 ± 1.87
	al	100 ± 0.00	96.07 ± 3.93	77.14 ± 6.98	95.36 ± 4.78	80.36 ± 7.76	98.21 ± 2.53
	av	99.29 ± 1.51	97.86 ± 2.50	53.57 ± 5.83	96.07 ± 3.13	56.43 ± 12.58	98.93 ± 2.41
	aw	99.64 ± 1.13	94.64 ± 9.41	55.36 ± 7.19	94.64 ± 5.89	63.57 ± 7.30	96.79 ± 2.64
	ay	98.57 ± 2.50	92.50 ± 10.71	64.64 ± 9.29	96.43 ± 3.37	52.14 ± 9.40	98.57 ± 2.50
	Ivb	99.52 ± 1.51	92.86 ± 4.63	69.52 ± 6.43	97.14 ± 2.46	59.52 ± 8.17	99.05 ± 2.01

In comparison, the MCSS + EEGNet benchmark achieved slightly lower performance (92.50%–98.57%), while the statistical‐only + SVM baseline showed substantially lower accuracy (53.57%–77.14%). The CSP + SVM baseline also demonstrated comparatively weaker results (52.14%–80.36%). Overall, the results indicate that the proposed MCSS + SVM framework consistently achieved the highest or near‐highest performance across all subjects and preprocessing settings.

Table 4 summarises the privacy robustness of the proposed MCSS + SVM framework under two adversarial threat models: likelihood‐based MIA and feature inversion attacks. Across all subjects, attack accuracy remained close to chance‐level (49.64%–50.36%), with corresponding AUC values ranging from 0.522 to 0.552, indicating minimal membership leakage. These results indicate that the attacker was unable to reliably distinguish whether a sample belonged to the training set, suggesting minimal membership leakage. Similarly, the feature inversion analysis demonstrated high reconstruction errors and low correlation values across all subjects, with reconstruction correlation ranging from 0.0215 to 0.2969. These low correlations indicate that the reconstructed signals do not closely resemble the original EEG representations, thereby supporting the non‐invertible and privacy‐preserving nature of the proposed framework.

**TABLE 4:** Privacy risk assessment of the proposed framework under MIA and feature inversion threat models.

	Likelihood of MIA attack		Feature inversion result	
Subject	Attack accuracy	Attack AUC	Mean reconstruction MSE	Mean reconstruction correlation
aa	50.36	0.531	36.27	0.0394
al	49.64	0.525	55.2012	0.0215
av	50.36	0.525	54.67	0.2248
aw	49.79	0.522	395.02	0.2733
ay	50	0.552	277.4278	0.2969
Ivb	49.9	0.547	94.37	0.2236

## 4 Discussion

The present study demonstrates that the proposed privacy‐preserving hybrid MCSS framework provides highly accurate and stable MI‐based EEG classification while substantially reducing information leakage risk. The superior performance of the framework is primarily attributable to its ability to model temporal state‐transition dynamics through the TPM‐based Markov representation, which preserves sequential neural activity patterns that are not captured by static statistical descriptors alone. In contrast, the statistical‐only baseline relies on distributional summaries such as mean and variance, which do not retain temporal dependencies and therefore show comparatively lower discriminative performance. The proposed MCSS + SVM framework consistently outperformed the comparative baselines across subjects, suggesting that the transition‐based representation provides a highly separable feature space for classification. Although the wavelet‐based preprocessing pipeline also produced strong results, the Butterworth‐based preprocessing generally yielded slightly superior performance, likely due to more effective preservation of MI‐relevant mu and beta oscillatory rhythms. Although only one deep learning architecture was evaluated, EEGNet was selected because it is a widely adopted EEG‐specific deep learning model and is commonly used as a benchmark architecture in EEG‐based BCI research. The superior performance of the MCSS + SVM framework compared with EEGNet may be attributed to several factors. First, the BCI Competition III datasets contain a relatively limited number of trials per subject, which may not be sufficient for deep neural networks to fully exploit their representation‐learning capacity. In contrast, SVMs are well known to perform effectively on small‐ to medium‐sized datasets and high‐dimensional feature spaces. Second, the proposed MCSS feature representation already provides a highly discriminative and compact abstraction of the EEG dynamics through TPMs and statistical descriptors. Consequently, the additional hierarchical feature‐learning capability of deep neural networks may offer limited benefit. Third, SVMs optimise a maximum‐margin decision boundary that is less prone to overfitting under limited training data, whereas deep learning models generally require substantially larger datasets to achieve stable generalisation. These findings suggest that, for handcrafted transition‐based representations such as MCSS, classical machine learning approaches may remain highly competitive and, in some cases, outperform more computationally intensive deep learning models. We acknowledge that additional deep learning architectures, such as DeepConvNet and ShallowConvNet, could provide further benchmarking. However, EEGNet was selected because it is one of the most widely adopted EEG‐specific deep learning architectures and serves as a representative deep learning baseline in EEG‐based BCI research.

Table 5 summarises the classification accuracy for each subject across competing approaches. The proposed MCSS + SVM consistently outperforms all comparative methods, achieving near‐perfect accuracy across subjects aa, al, av, aw, ay, and IVb. Specifically, the proposed framework attained accuracies of 99.5% for aa, 99.1% for al, 100% for av and ay, 99.21% for aw, and 99.52% for IVb, demonstrating excellent robustness and cross‐subject stability. In contrast, existing techniques show varying performance across subjects. For example, Moraes et al. (2024) [28] using IVAEI (Independent vector analysis + EEG inception), and Kabir et al. (2024) [29] with Relief‐F + LDA (Relief‐F + Linear discriminant analysis), reported lower accuracy scores, notably for av, where their models achieved only 69.3% and 75.36%, respectively. Similarly, although the multiobjective spatial‐temporal feature optimisation approach by Rezvani and Chaibakhsh (2024) [30] and the CSP–WPT–TL (common spatial patterns + wavelet packet transforms + transfer learning) method by Cai and Hong (2024) [31] delivered competitive accuracy, particularly on datasets al and aa, they fell short on datasets like av and aw compared to the proposed study.

**TABLE 5:** Comparison of our proposed method with existing studies that analysed the same datasets.

Authors	Methods + classifier	Accuracy					
		aa	al	av	aw	ay	IVb
The proposed Study	MC–SVM	99.5	99.1	100	99.21	100	99.52
Moraes et al. (2024) [28]	IVAEI	84.3	96.4	69.3	92.1	91.4	
Kabir et al. (2024) [29]	Relief‐F + LDA	89.29	98.57	75.36	97.51	96.43	
Sanaz Rezvani and Ali Chaibakhsh (2024) [30]	Multiobjective spatial‐temporal feature optimisation	91.98	100	81.07	94.64	93.25	
Miao Cai and Jie Hong (2024) [31]	(CSP + WPT)–TL	95.5	100	86.2	94.6	94.8	
Zhihui Li and Ming Meng (2024) [32]	MCSCA	84.31	89.70	76.92	88.23	100	
Thrikkannoor Kolathod and Sanjay (2023) [33]	covariance matrices + DNN	94.27	98.88	95.19	96.77	100	
Kabir et al. (2023) [34]	SRCFS + LDA	88.03	97.98	74.17	94.76	95.31	
Khanam et al. (2023) [10]	CSP–MKNN	97.3	100	90.3	92.4	95.6	
Yu et al. (2021) [35]	IEFD + WELCHPSD	99.52	99.35	98.89	99.52	100	93.19
Sadiq et al. (2020) [36]	MSPCA	95.22	98.63	92.91	72	100	99.52
Siuly and Li (2015) [20]	OA & NB‐based approach	100	100	90.91	90.91	95.45	86.36

Furthermore, deep learning‐based methods, such as the covariance matrix with DNN (deep neural network) by Thrikkannoor and Sanjay (2024) [33], also achieved high accuracy levels, such as 100% on ay, but did not maintain consistent performance across all subjects. Similarly, methods like SRCFS + LDA (subspace randomisation and collaboration‐based unsupervised feature selection + LDA) by Kabir et al. (2023) [34] and CSP–MKNN (CSP + medium KNN) by Khanam et al. (2023) [10] also achieved high accuracies on selected subjects but did not maintain consistent performance across all datasets. In earlier studies, Yu et al. (2021) [35] with IEFD + WELCHPSD (improved empirical Fourier decomposition + Welch power spectral density) and Sadiq et al. (2020) [36] with MSPCA (machine learning algorithm multiscale principal component analysis) also showed competitive performance but did not achieve the uniformly high accuracy demonstrated by MCSS+SVM.

A key strength of the proposed framework lies in its privacy‐preserving feature representation. The privacy evaluation further supports the practical value of the framework, with MIAs remaining close to the chance level and feature inversion attacks demonstrating low reconstruction similarity, indicating that the Markov‐derived features substantially reduce subject‐identifiable waveform information while maintaining classification performance. Unlike conventional privacy‐preserving frameworks such as federated learning, differential privacy, and encryption, where privacy is typically enforced through explicit noise injections or gradient perturbation that may reduce classification accuracy, the proposed MCSS framework adopts a fundamentally different mechanism [37, 38]. Privacy is achieved through non‐invertible feature abstraction rather than noise perturbation. Specifically, the raw EEG waveform is transformed into symbolic TPMs that preserve discriminative temporal dynamics while removing subject‐identifiable waveform morphology. Because no artificial noise is introduced into the discriminative feature space, the classification performance remains high. In fact, the TPM representation may further improve robustness by suppressing nuisance variability and subject‐specific amplitude fluctuations, thereby preserving or even enhancing class separability.

Despite these advantages, several limitations should be acknowledged. First, the proposed MC framework assumes memoryless transitions and stationary transition probabilities, which may not fully capture long‐range temporal dependencies and evolving nonlinear dynamics in EEG signals. Second, the study was conducted on benchmark datasets with a limited number of subjects using subject‐specific analyses, which may restrict broader generalisability to larger and more heterogeneous populations. Future studies should therefore evaluate the framework on larger multi‐subject cohorts and clinical EEG datasets and explore more advanced temporal and comparative architectures, including hidden Markov models, RNN‐enhanced Markov processes, transformer‐based models, FBCSP, and Riemannian geometry‐based classifiers under the same evaluation protocol.

## 5 Conclusion

In this study, we proposed a privacy‐preserving hybrid MCSS framework for MI‐based EEG classification, in which neural activity is modelled as transitions across six discrete symbolic amplitude states derived through amplitude‐based quantisation. By transforming spatially filtered EEG signals into symbolic state‐transition representations and encoding them as TPMs, the framework effectively captures meaningful spatio‐temporal dynamics while substantially reducing exposure of subject‐identifiable waveform information. The TPM‐based abstraction significantly reduces the ability to reconstruct the original EEG waveform, thereby enhancing privacy protection during data processing and storage. When combined with SVM classification, the proposed framework achieved consistently high classification performance across all subjects in the BCI Competition III Datasets IVa and IVb, demonstrating strong accuracy, stability, and robustness under a leakage‐safe evaluation protocol. Comparative analyses against multiple baseline classifiers, deep learning benchmarks, and alternative preprocessing strategies further confirmed the reliability and generalisability of the proposed MCSS+SVM framework.

The privacy robustness of the framework was further supported by two empirical threat‐model evaluations, in which MIAs remained close to chance level, and feature inversion attacks yielded low reconstruction similarity, indicating limited information leakage from the Markov‐derived features. Overall, the proposed MCSS+SVM framework provides a robust, accurate, and privacy‐preserving solution for scalable real‐world BCI applications.

## Author Contributions

**Taslima Khanam**: conceptualization, methodology, software, formal analysis, investigation, data curation, writing – original draft. **Siuly Siuly**: conceptualization, supervision, methodology, writing – review & editing. **Hua Wang**: supervision, validation, writing – review & editing.

## Funding

The authors have nothing to report.

## Conflicts of Interest

The authors declare no conflicts of interest.

## Data Availability

The datasets analysed during the current study are publicly available from the BCI Competition III repository and can be accessed through the original dataset source.
